# Supplementary material for: Clinical characteristics and outcomes of COVID-19 patients with diabetes mellitus in Kuwait
Source: Heliyon. 2021 Apr 5;7(4):e06706. doi: 10.1016/j.heliyon.2021.e06706 (PMC8020058; doi:10.1016/j.heliyon.2021.e06706)
Supplement: T2D_COVID_manuscript_FINAL [file mmc1.docx]

**Supplementary**

**Figure 1. Cohort age structure stratified by diabetes status. On average,** non-diabetic group had a mean age of 39.55 (± 16.59) years while diabetic group had a mean age of 56.44 (± 11.64) years (p-value <0.001, student’s T test).
